# Implementation of Sensitive Method for Determination of Benzophenone and Camphor UV Filters in Human Urine

**DOI:** 10.3390/toxics12120837

**Published:** 2024-11-21

**Authors:** Veronika Gomersall, Katerina Ciglova, Jana Pulkrabova

**Affiliations:** Department of Food Analysis and Nutrition, Faculty of Food and Biochemical Technology, University of Chemistry and Technology, Prague, Technicka 3, 166 28 Prague, Czech Republic; veronika.vondraskova@vscht.cz (V.G.); katerina.urbancova@vscht.cz (K.C.)

**Keywords:** biomonitoring, UHPLC-MS/MS, urine analysis, UV filters

## Abstract

The level of the human body’s burden of benzophenone and camphor ultraviolet (UV) filters can be estimated from their urinary levels. The present study describes the implementations and validation of the sensitive analytical method for the analysis of seven benzophenone and two camphor UV filters in urine. Sample preparation includes overnight enzymatic hydrolysis and ethyl acetate extraction followed by purification by dispersive solid-phase extraction using a sorbent Z-Sep. For the analysis, ultra-high performance liquid chromatography coupled with tandem mass spectrometry was used. Validation was performed using a Standard Reference Material^®^ 3673 and an artificially contaminated urine sample. Target analyte recoveries ranged from 79–113% with repeatability expressed as a relative standard deviation of 2–15%. The limits of quantification were between 0.001 and 0.100 ng/mL in urine. This method was subsequently applied to examine the urine samples collected from Czech women. The analytes benzophenone-1 and 4-hydroxy-benzophenone were the most common analytes present in 100% of the samples, whereas benzophenone-3 was quantified in only 90% of the urine samples. The other four determined benzophenone derivatives were quantified in ≤33% of the samples. The derivatives of camphor were not detected in any samples. This method could be applied in biomonitoring studies.

## 1. Introduction

Human biomonitoring is an important tool for assessing human exposure to various chemicals. As part of the European Human Biomonitoring Initiative (HBM4EU—Human Biomonitoring for Europe), a list of 18 priority groups of chemicals to focus on has been established. This list includes environmental contaminants such as polycyclic aromatic hydrocarbons (PAHs), flame retardants, bisphenols or phthalates and di-iso-nonyl cyclohexane-1,2-dicarboxylate (DINCH), which has attracted attention for several years [1]. Recently, the group of benzophenone and camphor UV filters has been added to this list. Specific compounds of interest to HBM4EU are benzophenone (BP), benzophenone-1 (BP-1), benzophenone-2 (BP-2), benzophenone-3 (BP-3), 3-(4-methylbenzylidene)-camphor (4-MBC), 3-benzylidene camphor (3-BC), 4-hydroxy- benzophenone (4-OH-BP) and 4-methyl-benzophenone (4-MBP) [2].

Compounds from the group of ultraviolet (UV) filters are characterised by their ability to absorb UV radiation. Therefore, they are an essential component of sunscreens, but also of personal care products, to protect human skin from dangerous UV radiation such as UVA (315–400 nm) and UVB (280–315 nm) [3]. The use of benzophenone and camphor UV filters is not only associated with the cosmetics industry, but due to their properties they are also used in the manufacturing of products where UV damage is undesirable (plastic products, food packaging or textiles) or as photo-initiators (inks) [4,5,6,7].

Due to the widespread use of benzophenone and camphor UV filters, personal exposure to these compounds could be caused by a variety of sources including the use of cosmetic products [8,9], the digestion of contaminated food or water [10,11] or the inhalation of air containing dust particles contaminated with these compounds [12]. Human exposure to these contaminants is a concern due to their potential impact on human health. Some benzophenone and camphor UV filters are considered potential endocrine disruptors [13,14,15] and possibly carcinogenic to humans (Group 2B) according to the Internal Agency for Research on Cancer (IARC) [16].

Knowledge of the metabolism of these compounds is limited and biotransformation products have been studied for the compounds BP, BP-3 [17], BP-2 [18] and 4-MBC [19]. After a person is exposed to them, these compounds enter the circulatory system and can be partially metabolised. The biotransformation process takes place predominantly in the liver through the action of cytochrome P450. In the first phase of metabolism, the parent compound is converted into its metabolites by hydrolysis and hydroxylation. The conjugation reactions of the parent compound and/or its metabolites with endogenous compounds such as glucuronic or sulphuric acid take place in the second phase of metabolism [20,21]. Thereafter, benzophenone and camphor UV filters and their metabolites are excreted in a free or conjugated form in urine or faeces [22,23,24].

To monitor the level of human exposure to benzophenone and camphor UV filters, the most commonly published approach is the analysis of urine, in which the free and/or conjugated parent compounds and their metabolites are monitored. In the Ye et al. study, of the percentages of BP-3 identified within the urine samples, BP-3 free, glucuronide and sulphate were 3, 85 and 12%, respectively [25]. Therefore, the first step of the urine sample preparation requires hydrolysis to dissolve their conjugated forms with glucuronic and/or sulphuric acid. The enzyme β-glucuronidase from *Helix pomatia* is the most commonly used because of its glucuronidase and sulfatase activity. A commonly used isolation technique is multiple liquid–liquid extraction (LLE) using ethyl acetate as the extraction solvent [26,27,28] or a mixed solvent of ethyl acetate and methyl *tert*-butyl ether (1:1 or 1:5, *v*/*v*) [29,30]. Alternatively, the solid–phase extraction (SPE) isolation technique using cartridges containing sorbents such as Envi-18 [31], VBond Elut-C18 [32] or Oasis HLB [33] is used. In most published studies, liquid chromatography (LC) coupled with tandem mass spectrometry (MS/MS) and electrospray ionisation (ESI) [34,35,36] or atmospheric-pressure chemical ionisation (APCI) [32,37] are used to identify and quantify benzophenone and camphor UV filters.

Since 2011, published biomonitoring studies have mainly focused on determining the concentrations of BP-3 and its two metabolites, BP-1 and benzophenone-8 (BP-8) and BP-2 and 4-OH-BP in urine [38,39,40,41,42]. In addition to the benzophenone UV filters mentioned above, concentrations of benzophenone-6 (BP-6) were assessed in a study focused on the female population in Tunisia [43]. The most abundant spectrum of benzophenone and camphor UV filters was determined in Danish studies [37,44], in which the six compounds mentioned above were determined in urine in addition to BP, benzophenone-7 (BP-7), 4-MBP, 4-MBC and 3-BC. The concentration of camphor UV filters (4-MBC and/or 3-BC) in urine was also assessed in Chinese studies [11,45].

This study was aimed at implementing and validating a new method for the determination of seven benzophenones (BP-1, BP-2, BP-3, BP-6, BP-7, BP-8 and 4-OH-BP) and two camphor UV filters (4-MBC and 3-BC) in urine by ultra-high performance liquid chromatography coupled with tandem mass spectrometry (UHPLC-MS/MS). Two extraction methods were tested for the isolation of the target compounds. This approach was based upon our earlier experience with urine matrix from the implementation of methods for the evaluation of monohydroxylated metabolites of polycyclic aromatic hydrocarbons (OH-PAHs) [46] and the metabolites of phthalates and DINCH [47].

## 2. Materials and Methods

### 2.1. Certified Standards, Chemicals and Other Materials

The list of certified standards used and their purity and manufactures are given in Table S1. All individual standards, mixtures of standards and mixtures of isotopically labelled standards were prepared in methanol. All solutions were stored at −20 °C in the freezer. The Standard Reference Material^®^ (SRM) 3673 used for the validation experiments was obtained from the NIST (Gaithersburg, MD, USA). Acetic acid (99% p.a.) was supplied by Penta a.s. (Prague, Czech Republic). Methanol (HPLC gradient) and anhydrous magnesium sulphate (for analysis) were supplied by Merck KGaA (Darmstadt, Germany). Ethyl acetate (Chromasolv™) and formic acid (>98%) were purchased from Honeywell (Charlotte, NC, USA). The enzyme β-glucuronidase (type HP-2, glucuronidase activity ≥ 100,000 units/mL, sulfatase activity ≤ 7500 units/mL) and the sorbent SupelTM QuE Z-Sep were purchased from Sigma Aldrich (Darmstadt, Germany). Sodium hydroxide (99.9% p.a.) was purchased from Lach-Ner, s.r.o. (Neratovice, Czech Republic). MicroSpin centrifuge tube filters (polyvinylidene fluoride (PVDF), pore size 0.2 µm) were bought from Ciro Manufacturing Corporation (Deerfield Beach, FL, USA) and 50 mL polypropylene centrifuge tubes from Kartell S.p.A (Noviglio, Italy).

### 2.2. Sample Collection

The 30 samples used in this pilot study were provided by the Institute of Experimental Medicine, Academy of Sciences of the Czech Republic in Prague as part of the project ‘QUALITAS’—Wellbeing in Health and Disease. These samples were originally collected for the analysis of OH-PAHs. The study was approved by the Ethics Committee of the hospital Ceske Budejovice (reference number 5/16). Each participant signed a written consent form and completed a questionnaire on age, body mass index (BMI), lifestyle and residence. The samples analysed in this study were collected from women living in the Czech city of Ceske Budejovice in the south of the Czech Republic. Of the 30 samples analysed, 15 were collected in summer (July–August 2016) and 15 in winter (January–February 2017) from 30 separate individuals. All urine samples were stored in the freezer at −20 °C before analysis.

### 2.3. Description of Tested Methods

#### 2.3.1. “Dilute-and-Shoot”

The first method tested was originally developed for the analysis of 18 phthalate and four DINCH metabolites in urine [47]. This method starts with enzymatic hydrolysis to break the bond between the target analyte and glucuronic acid and/or sulphate. Isotopically labelled standards solution at a concentration of 10 ng/mL in a vial, 0.04 mL acetate buffer (pH 5) and 0.02 mL enzyme β-glucuronidase are added to 0.3 mL urine samples. The enzymatic hydrolysis then runs overnight (approximately 16 h) at 37 °C. To the hydrolysed sample, 0.02 mL formic acid is added to stop the enzymatic hydrolysis together with 0.02 mL methanol. The prepared sample is then centrifuged (5000 rpm, 4 °C) for 30 min to sediment the solid particles at the bottom of the tube. The supernatant is then transferred to a vial for instrumental analysis by UHPLC-MS/MS.

#### 2.3.2. LLE Followed by a Clean-Up with Dispersive Solid-Phase Extraction (d-SPE)

The second method tested was originally developed for the determination of OH-PAHs in urine [46]. This method also starts with enzymatic hydrolysis. Isotopically labelled standards solution at a concentration of 10 ng/mL in a vial, 10 mL acetate buffer (pH 5) and 0.02 mL enzyme β-glucuronidase are added to 5 mL urine samples. The enzymatic hydrolysis then runs overnight (approximately 16 h) at 37 °C. To the hydrolysed sample, 15 mL ethyl acetate is added. The prepared mixture is shaken vigorously for 1 min and then centrifuged (5 min, 10,000 rpm). Then, 12 mL of the upper layer is transferred to a tube into which 0.18 g sorbent Z-Sep and 1.8 g anhydrous MgSO_4_ were previously weighed. This mixture is shaken for a further 1 min and centrifuged (5 min, 10,000 rpm). A total of 8 mL of the supernatant is then transferred to a pear-shaped flask and evaporated (200 mbar, 40 °C) to the last drop, then dried under a gentle stream of nitrogen. The evaporated sample is then reconstituted in 0.25 mL methanol. To remove residues of the sorbent used, the reconstituted sample is filtered with micro-filters (PVDF, 0.2 µm). The filtered sample is then transferred to a vial for instrumental analysis by UHPLC-MS/MS.

### 2.4. Instrumental Analysis

Target compounds were analysed using the Agilent Technologies (Santa Clara, CA, USA) 1290 Infinity II UHPLC system equipped with the Agilent 1290 Infinity II Vialsampler G7129B autosampler, sampler cooler G7129B, binary pump Agilent 1290 Infinity II High Speed Pump 7120A and heated column holder Agilent 1290 Infinity II ICC Column heater G7129B. The analytes were separated on a Kinetex Core-Shell Pentafluorophenyl (PFP) column measuring 100 mm × 2.1 mm × 1.7 µm, Phenomenex (Torrance, CA, USA) at 40 °C. The mobile phase consisted of (A) water and (B) methanol using a gradient with an initial mobile phase composition of 90% (A) and 10% (B) at a flow rate of 0.3 mL/min followed by the change of composition after 0.5 min to 60% (A) and 40% (B) at a flow rate of 0.35 mL/min and after 11 min with composition 0% (A) and 100% (B) and a flow rate of 0.4 mL/min. Then, the composition was kept isocratic until 12 min, when the flow rate was increased to 0.45 mL/min; finally, from 12.2 to 13 min, reconditioning of the column took place at a flow rate of 0.5 mL/min. The injected volume of the sample was 5 µL and the autosampler was kept at 10 °C. The UHPLC was coupled to an MS/MS system QTRAP^®^ 6500+, Sciex (Concord, ON, Canada). Target substances were ionised using the electrospray ion source IonDrive Turbo V operated in both positive and negative ion mode with a capillary voltage of ±4500 V, desolvation temperature of 500 °C, curtain gas of 40 psi and nebuliser and turbo gases of 55 psi. The unit was operated in multiple reaction monitoring (MRM) mode.

### 2.5. Quality Assurance/Quality Control, Validation and Matrix Effects

The validation was performed in accordance with the European Commission’s guidance document “Analytical quality control and method validation procedures for pesticide residues analysis in food and feed” [48]. The final analytical method (LLE with d-SPE) was validated with a previously tested blank urine sample artificially contaminated with the target analytes at three concentrations—0.005, 0.05 and 0.5 ng/mL in urine (all concentrations were prepared in six replicates) and with the SRM 3673 in six replicates. The SRM 3673 was only used for the validation of BP-3, as it was the only compound certified in this material from the range of analytes. Concentrations of target compounds were quantified using a solvent calibration in the range of 0.01–100 ng/mL methanol with internal standards at a level of 10 ng/mL.

A blank process sample (deionised water instead of urine) was also prepared during the validation experiments. The measured contamination was then subtracted from the tested urine samples. The limits of quantification (LOQs) were determined as the lowest calibration values at which the signal-to-noise ratio (S/N) was >10. The measured concentrations of the target analytes were corrected with available isotopically labelled analogues. Matrix effects for each target analyte were calculated according to Equation (1).

(1) $$Matrix\;effect=(\frac{{AREA}_{matrix+\;standard}\;-{AREA}_{\;blank\;matrix}}{{AREA}_{solvent\;+\;standard}\;}-1)\times 100$$

## 3. Results and Discussion

### 3.1. UHPLC-MS/MS Method Development

In order to achieve sufficient sensitivity for the precise measurement of the concentrations of the target analytes in the urine samples (<0.01–4.61 ng/mL in urine [38,44,49,50]), the following parameters of the UHPLC-MS/MS method were tested: (i) the ionisation and MS/MS parameters, (ii) the mobile phase composition and (iii) the type of chromatographic columns and elution gradient.

**Figure 1:**
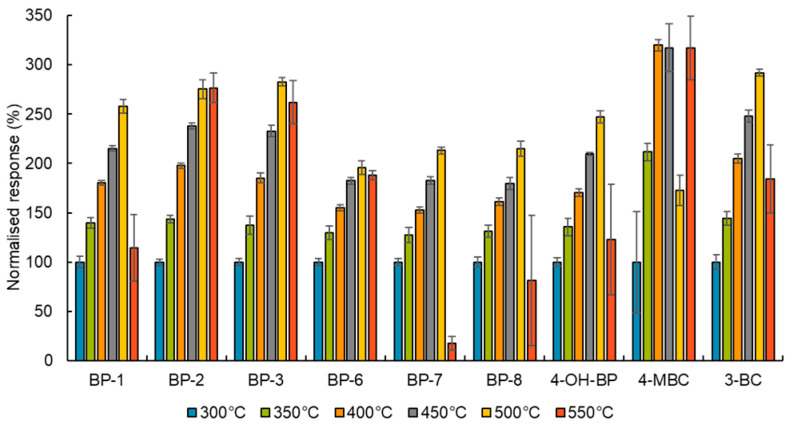
The influence of desolvation temperature on analyte response. Note: The desolvation temperature was tested with a capillary voltage set at ±4500 V. The data were normalised to analyte response at 500 °C. The analyte response was calculated from the qualifications transition from a standard solution of 10 ng/mL of the target analytes in methanol injected six times. The error bars correspond to relative standard deviations.

**Figure 2:**
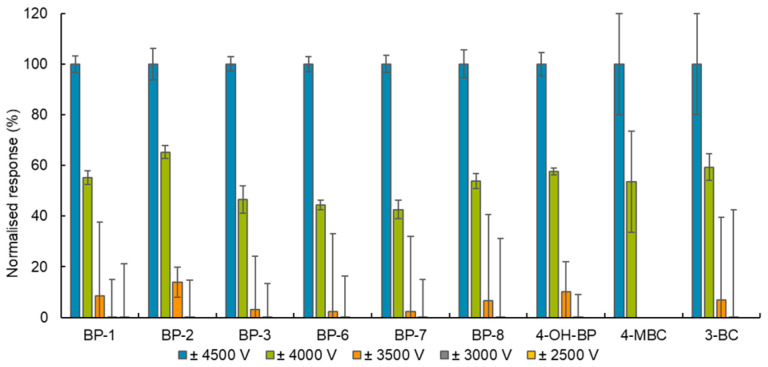
The influence of capillary voltage on analyte response. Note: The capillary voltage was tested with a desolvation temperature set at 500 °C. The data were normalised to analyte response at ±4500 V. The analyte response was calculated from the qualifications transition from a standard solution of 10 ng/mL of the target analytes in methanol injected six times. The error bars correspond to relative standard deviations.

**Figure 3:**
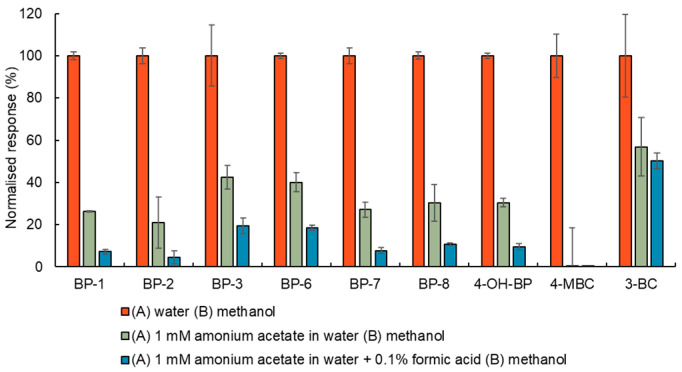
The influence of mobile phase on analyte response. Note: The data were normalised to analyte response with (A) water and (B) methanol. The analyte response was calculated from the qualifications transition from a standard solution of 10 ng/mL of the target analytes in methanol injected six times. The error bars correspond to relative standard deviations.

**Figure 4:**
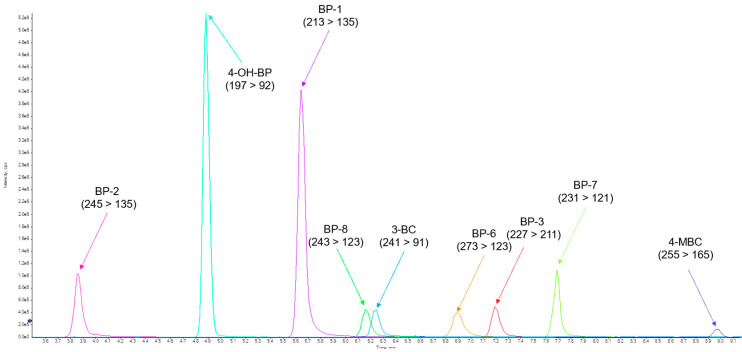
Extracted-ion chromatogram (XIC) of standard mixture of target analytes at concentration 10 ng/mL in methanol (HSS T3 C18 (100 mm × 2.1 mm × 1.8 µm) from Waters (Milford, MA, USA)).

**Figure 5:**
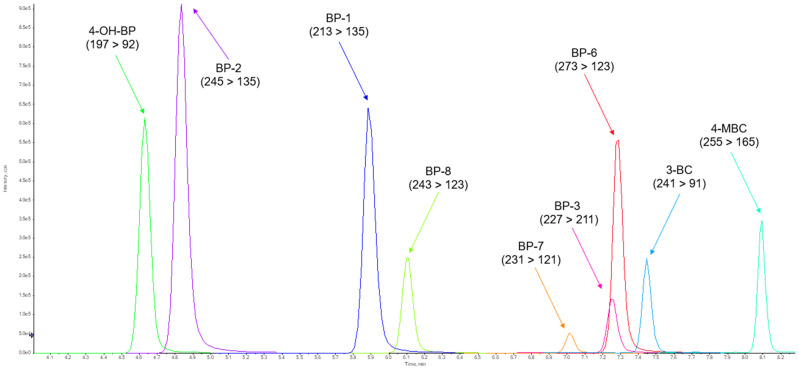
Extracted-ion chromatogram (XIC) of standard mixture of target analytes at concentration 10 ng/mL in methanol (PFP Kinetex (100 mm × 2.1 mm × 1.7 µm) from Phenomenex (Torrance, CA, USA)).

(i) The highest intensity MRM transitions and their values for collision energy, declustering potential, entrance potential and cell exit potential for each target compound were obtained by infusing a standard solution of the target analytes of 100 ng/mL in methanol directly into the ESI in positive and negative mode followed by automatic tuning (see Table S2 in the Supplementary Data). To obtain the optimal value for the ionisation parameters, capillary voltage and desolvation temperature, the responses of a standard solution of the target analytes 10 ng/mL in methanol (injected six times) were evaluated for several adjusted values of these parameters. As Figure 1 and Figure 2 show, the peaks with the highest intensity were obtained when the capillary voltage and desolvation temperature were set to ±4500 V and 500 °C, respectively.
(ii) The influence of the mobile phase composition on the ionisation efficiency and peak shape of the target analytes were also investigated (see Figure 3).
  The addition of mobile phase modifiers resulted in a reduction in analyte response of about 43–100%, so pure (A) water and (B) methanol were used in the final method. This composition also gave Gaussian peaks (see Figure 4 and Figure 5).
(iii) Based on the literature review, an HSS T3 C18 (100 mm × 2.1 mm × 1.8 µm) reversed-phase column from Waters (Milford, MA, USA) was selected for the separation of the target analytes. The tested settings for the gradient elution programme are listed in Table 1.

Initially, the composition of the mobile phase was always 10% methanol, changing to 10–40% methanol during the 0.5 min gradient. The time settings for the 40–100% methanol gradient and an isocratic hold at 100% methanol were changed according to the retention time of the first and last analyte, as the target analytes are not isomers and there was no need for their complete separation. The shape of the peaks was similar in all of the gradient elution programmes tested; therefore, the gradient elution programme III with a total run time of 13 min was chosen as the final programme to obtain an efficient analysis of a larger number of samples.

Figure 4 shows the extracted-ion chromatogram (XIC) of the standard solution of the target analytes at 10 ng/mL in methanol. The second chromatography column tested was PFP Kinetex (100 mm × 2.1 mm × 1.7 µm) from Phenomenex (Torrance, CA, USA) with the gradient III programme. The extracted-ion chromatogram (XIC) of the standard solution of 10 ng/mL of the target analytes in methanol is shown in Figure 5. Sufficient sensitivity and a Gaussian shape of the peak were achieved with both chromatographic columns.

### 3.2. Extraction Method Development

**Figure 6:**
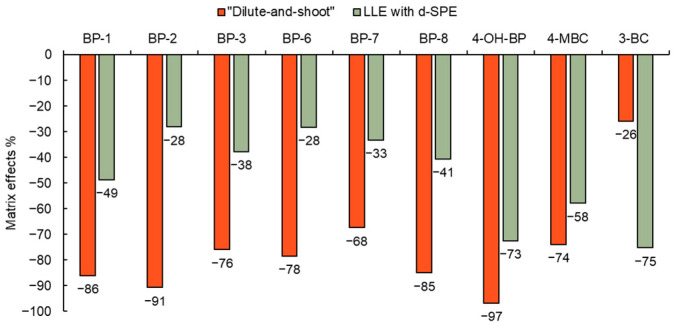
Influence of urine matrix on ionisation of analytes.

Two extraction methods (“dilute-and-shoot” and LLE with d-SPE) were tested for the isolation of the target compounds. The evaluation of the matrix effects in both extracts was performed. As shown in Figure 6, signal suppression was observed for all target analytes; therefore, isotopically labelled standards were used to correct for these losses. Analytes for which their deuterated forms were not available were corrected using the isotopically labelled standards of the closest analyte chromatographically.

The extraction methods were tested on an unhydrolyzed sample of the previously tested blank urine to avoid dissolving the conjugated forms of benzophenone and camphor from the UV filters. The six replicates of urine for each technique were artificially contaminated with the target analytes to a concentration of 1 ng/mL in urine. For both extraction methods, the blank process sample was analysed simultaneously. The LOQ, recovery and repeatability, expressed as relative standard deviation (RSD), for the tested methods are shown in Table 2.

#### 3.2.1. “Dilute-and-Shoot”

Since the urine sample was only diluted and not further purified and the urine pigments and other matrix components were present in the extract and caused the matrix effects during ionisation of the analytes, the signal suppression of the target compounds was 26–97% (see Figure 6). As shown in Table 2, the LOQs of the target analytes for this method ranged from 0.01–0.07 ng/mL in urine. The recovery (corrected to isotopically labelled standards) for six out of nine target analytes was in the range of 70–120% with an RSD ≤ 20% [48]. The “dilute-and-shoot” isolation technique is very effective in terms of time and labour as it allows the processing of up to 40 urine samples per day. Another advantage of this simple method is the minimal use of plastic material and laboratory equipment, which could be possible sources of contamination in the laboratory.

#### 3.2.2. LLE with d-SPE

A second isolation technique, LLE using d-SPE as a purification step, tested whether the sorbent binds the analytes, and no significant binding of the target analytes to the sorbent was observed. Signal suppression of the target analytes due to matrix effects was lower for eight out of nine analytes for the urine extract obtained by this method compared to the “dilute-and-shoot” method (see Figure 6). As shown in Table 2, the LOQs of the target substances for this method were in the range of 0.001–0.005 ng/mL in urine and were about one order of magnitude lower than for the “dilute-and-shoot” method. The recovery (corrected to isotopically labelled standards) was in the range of 70–120% for eight of the nine target analytes with an RSD ≤ 20% [48]. Due to the lower LOQs achieved, the second extraction method tested, LLE with d-SPE, was selected for validation in the next step.

### 3.3. Method Validation

The sample preparation and optimised conditions of the instrumental method for the determination of benzophenone and camphor UV filters (when the PFP Kinetex column was used for separation of the analytes) were identical to the sample preparation and conditions of the instrumental method for the determination of OH-PAHs in urine that is routinely used in our department. As such, the validation process also included 11 analytes from the group of OH-PAHs to allow the simultaneous determination of OH-PAHs and UV filters from a urine extract in one analytical run.

Method validation (LLE followed by d-SPE) was performed using SRM 3673 with a certified value for BP-3 and 9 OH-PAHs and an enriched blank urine sample at three concentrations (0.005, 0.05 and 0.5 ng/mL in urine) for the remaining target compounds, which were not certified in this material.

In the following paragraph, only the results for seven benzophenone and two camphor UV filters are discussed. For the performance characteristics of the analytical method for 11 OH-PAHs, see Tables S3 and S4.

The measured concentration of BP-3 in SRM 3673 (263 ± 13 ng/mL in urine) was slightly lower than the certified value (279 ± 7 ng/mL in urine), but still with an acceptable recovery of 94% and an RSD of 5% (see Table 3).

At the 0.005 ng/mL in urine validation level, six out of ten analytes (BP-1, BP-2, BP-6, BP-7, BP-8 and 4-OH-BP) were successfully validated with recoveries ranging from 79–111% and an RSD of 4–9%. At the 0.05 ng/mL in urine validation level, six out of ten analytes (BP-1, BP-2, BP-6, BP-7, BP-8 and 4-OH-BP) were successfully validated with recoveries ranging from 85–110% and an RSD of 8–15%. At the 0.5 ng/mL in urine validation level, eight out of ten analytes (additionally, 4-MBC and 3-BC) were validated with recoveries in the range of 95–113% and an RSD of 2–6% (see Table 4).

The analytes 4-MBC and 3-BC failed only at the lower validation level due to lower sensitivity. The analytes found in the blank process samples were BP-3, BP-6 and 4-OH-BP, but in concentrations that can be considered trace values and subtracted from the actual concentrations in the urine sample. The same problem was mentioned in the study by Gao et al. [39] in which the blank process samples contained the analytes BP-3 (0.39 ng/mL) and 4-OH-BP (0.05 ng/mL).

Table 5 summarises the selected methods used in published biomonitoring studies involving the determination of a broader range of benzophenone and camphor UV filters in urine. In the present study, the achieved LOQ for BP-3 (0.1 ng/mL) was ten times higher than in the method published by Song et al. [27] (0.01 ng/mL), but comparable to values obtained by other methods summarised. The LOQs for the analytes BP-2, BP-6, BP-7, BP-8 and 4-OH-BP (0.001–0.005 ng/mL) were at least 2–100 times lower than the selected methods. The same LOQ for BP-2 (0.002 ng/mL) was achieved compared to the method of Lu et al. [9].

### 3.4. Method Applicability

The final validated method was used in a pilot study to evaluate the concentrations of seven benzophenone and two camphor UV filters in the urine of Czech women (*n* = 30) collected during the summer and winter seasons. The analytes BP-1 and 4-OH-BP, metabolites of BP-3 and BP, respectively, were the most abundant analytes, present in 100% of the samples, while BP-3 was quantified in only 90% of the urine samples. The other four benzophenone derivatives were quantified in <50% of the samples. The camphor derivatives 4-MBC and 3-BC were not detected in any sample. The measured concentrations of all target analytes ranged from <0.001–1340 ng/mL in urine. Summary information on the measured concentrations of all target analytes is in Table 6.

Exposure of the tested Czech women to BP-3 (median 18.0 ng/mL in urine), the most commonly studied compound from the benzophenone UV filter group, was twice as high as in the Chinese women (median 8.96 ng/mL in urine) studied by Gu et al. [33]. Compared to other European (Spanish and Danish) female populations (median 3.4 and 3.7 ng/mL in urine), the exposure of women from this study was five to six times higher [49,50]. Since the compounds BP-1 and BP-8 are known metabolites of BP-3, the correlation between these compounds was determined by calculating the Spearman correlation coefficient (*r_s_*). A high correlation was found between the measured concentrations of BP-3 and BP-1 (*r_s_* = 0.864) and a moderate correlation for BP-3 and BP-8 (*r_s_* = 0.546). The measured concentration of 4-OH-BP in the urine of the Czech women studied (median 0.351 ng/mL) was almost two times lower than in the urine of Chinese women (median 0.670 ng/mL in urine) [27] and one and a half times higher than in the urine of Danish women (median < 0.230 ng/mL adjusted urine) [51]. As mentioned above, the other benzophenone derivatives (BP-2, BP-6, BP-7 and BP-8) were only quantified in some of the urine samples (20, 7, 33 and 20% positive samples, respectively). This could indicate that exposure to these substances is very individual (frequency of use of cosmetic products, dietary habits). No statistically significant differences (*p* < 0.05) were found between the measured concentrations of benzophenone and camphor UV filters in urine samples collected in winter and summer. Our pilot study has several limitations. In order to reach a conclusion regarding benzophenone and camphor UV filter exposure in the Czech female population, it would be necessary to examine a larger number of samples and correlate the results with the concentration of creatine to eliminate differences between the hydration status of individual women. Moreover, the questionnaires did not contain the key information to assess the possible sources of exposure, such as the use of daily skin care products. As a result, no clear trend or possible source of exposure could be identified.

## 4. Conclusions

This method has all of the essential factors to be applicable in biomonitoring studies as it is very effective in terms of time and labour consumption, allowing for the processing of up to 30 samples of urine per day; it can be used for the simultaneous determination of a broad spectrum of two groups of emerging environmental contaminants (seven benzophenone and two camphor UV filters and eleven metabolites of PAHs). Sample preparation includes overnight enzymatic hydrolysis and LLE with ethyl acetate as the extraction solvent, followed by purification by d-SPE with the sorbent Z-Sep. Separation and quantification of the target analytes is performed using the UHPLC-MS/MS system. The assessment of human exposure to two different groups of environmental contaminants can be performed from a urine extract in one analytical run (15 min). This method was subsequently applied for the analysis of urine samples collected from a Czech female population (*n* = 30). The analytes BP-1 and 4-OH-BP were quantified in 100% of the urine samples, while BP-3 was quantified in only 90% of the urine samples. The other four determined benzophenone derivatives (BP-2, BP-6, BP-7 and BP-8) were quantified in ≤33% of the samples. The derivatives of camphor were not detected in any sample.

## Figures and Tables

**Table 1:** Tested gradients of mobile phase on column HSS T3 C18.

Name	Time (Min)	Flow (mL/min)	Composition of Mobile Phase (%)	
			Water (A)	Methanol (B)
Gradient elution programme I	0.0	0.30	90	10
	0.5	0.35	60	40
	10.0	0.40	0	100
	14.0	0.40	0	100
	14.1	0.40	90	10
	16.0	0.50	90	10
Gradient elution programme II	0.0	0.30	90	10
	0.5	0.35	60	40
	10.0	0.40	0	100
	12.0	0.40	0	100
	12.1	0.40	90	10
	14.0	0.50	90	10
Gradient elution programme III (final)	0.0	0.30	90	10
	0.5	0.35	60	40
	11.0	0.40	0	100
	12.0	0.45	0	100
	12.2	0.50	90	10
	13.0	0.50	90	10

**Table 2:** Comparison of the tested extraction methods.

Analyte	ISTD	“Dilute-and-Shoot”			LLE with d-SPE		
		LOQ	Recovery	RSD	LOQ	Recovery	RSD
		(ng/mL in urine)	(%)	(%)	(ng/mL in urine)	(%)	(%)
BP-1	4-OH-BP-d_4_	0.03	175	16	0.002	97	4
BP-2	BP-8-d_4_	0.03	79	5	0.002	90	10
BP-3	BP-3-d_3_	0.07	96	4	0.005	113	9
BP-6	BP-8-d_3_	0.01	66	5	0.001	98	11
BP-7	BP-8-d_3_	0.03	131	4	0.002	127	8
BP-8	BP-8-d_3_	0.07	120	2	0.005	116	3
4-OH-BP	4-OH-BP-d_4_	0.01	85	3	0.001	99	4
4-MBC	4-MBC-d_4_	0.07	94	2	0.005	102	5
3-BC	4-MBC-d_4_	0.07	72	5	0.005	97	3

Note: ISTD—isotopically labelled standards used to correct the possible matrix effects and/or extraction loses. The urine samples were artificially contaminated (six replicates for each technique) to a concentration of 1 ng/mL in urine for all target analytes.

**Table 3:** Performance characteristics of analytical method for analyte BP-3-LLE with d-SPE.

ISTD	BP-3-d_3_
LOQ ng/mL in urine	0.100
SRM 3673	
Certified value (ng/mL in urine)	279 ± 7
Measured value (ng/mL in urine)	263 ± 13 ^a^
Recovery (%)	92
RSD (%)	2

Note: ^a^ uncertainty was calculated as a standard deviation; ISTD—isotopically labelled standards used as surrogates.

**Table 4:** Performance characteristics of LC-MS/MS for target analytes (except BP-3)-LLE with d-SPE.

	BP-1	BP-2	BP-6	BP-7	BP-8	4-OH-BP	4-MBC	3-BC
ISTD	4-OH-BP-d_4_	BP-8-d_4_	BP-8-d_3_	BP-8-d_3_	BP-8-d_3_	4-OH-BP-d_4_	4-MBC-d_4_	4-MBC-d_4_
LOQ ng/mL in urine	0.002	0.002	0.001	0.002	0.005	0.001	0.100	0.100
Level 0.005 ng/mL in urine								
Recovery (%)	91	79	97	95	101	111	-	-
RSD (%)	4	9	8	4	8	9	-	-
Level 0.05 ng/mL in urine								
Recovery (%)	101	85	102	105	107	110	-	-
RSD (%)	8	15	12	9	10	13	-	-
Level 0.5 ng/mL in urine								
Recovery (%)	99	95	100	101	98	106	113	101
RSD (%)	3	5	2	5	2	6	3	3

Note: ISTD—isotopically labelled standards used as surrogates; LOQ—limit of quantification; RSD—repeatability expressed as relative standard deviation.

**Table 5:** Summary of selected methods for the determination of benzophenone and camphor UV filters in urine used in published biomonitoring studies.

Analytes	Volume of Sample	Preparation Step	Instrumental Method	LOD (ng/mL)	LOQ (ng/mL)	Ref.
BP	0.1 mL	Enzymatic hydrolysis		2.4	*7.2*	[37]
BP-1		β-glucuronidase/sulfatase	HPLC-APCI (+/−)-MS/MS	0.3	*0.9*	
BP-2		1 M acetate buffer (pH 5.5)	column Hypersil Gold aQ	0.4	*1.2*	
BP-3		incubation 37 °C, 1.5 h	(50 mm × 4 mm × 3.0 µm)	0.3	*0.9*	
BP-7				0.4	*1.2*	
4-OH-BP		online-Turbo-Flow column		0.2	*0.6*	
4-MBP		TurboFlow Cyclone P		0.5	*1.5*	
4-MBC		(50 mm × 0.5 mm)		0.9	*2.7*	
3-BC				1.0	*3.0*	
BP-1	2 mL	Enzymatic hydrolysis		0.5	*1.5*	[45]
BP-2		β-glucuronidase-*Helix pomatia*	UHPLC-ESI(+)-MS/MS	0.95	*2.9*	
BP-3		1 M acetate buffer (pH 5.5)	column UPLC BEH 18	0.08	*0.24*	
4-OH-BP		incubation 37 °C, overnight	(100 mm × 2.1 mm × 1.7 µm)	0.28	*0.84*	
BP-8		SPE		0.31	*0.93*	
4-MBC		cartridge ABS ELUT-Nexus		0.55	*1.7*	
3-BC		(60 mg/3 mL, Agilent Technologies)		0.26	*0.78*	
BP-1	2 mL	Enzymatic hydrolysis		0.01	*0.03*	[11]
BP-2		β-glucuronidase/sulfatase	HPLC-ESI(+)-MS/MS	0.01	*0.03*	
BP-3		1 M acetate buffer (pH 5.0)	column Eclipse plus C18	0.01	*0.03*	
BP-4		incubation 37 °C, 12 h	(n.s.)	0.005	*0.02*	
BP-8		LLE		0.01	*0.03*	
4-MBC		acetone:trichlormethane (1:1, *v*/*v*)		2.5	*7.5*	
BP-1	2 mL urine	Enzymatic hydrolysis			0.02	[27]
BP-3		β-glucuronidase/sulfatase	HPLC-ESI(-)-MS/MS		0.01	
BP-4		1 M acetate buffer (pH n.s.)	column Betasil C18		0.02	
BP-6		incubation 37 °C, 12 h	(150 mm × 2.1 mm × 3.5 µm)		0.02	
BP-7			Javelin guard column Betasil C18		0.20	
BP-8		LLE	(20 mm × 2.1 mm × 5 µm)		0.01	
BP-9		ethyl acetate			0.01	
4-OH-BP					0.02	
BP-1	2 mL urine	Enzymatic hydrolysis			0.002	[9]
BP-2		β-glucuronidase/sulfatase	HPLC-ESI(-)-MS/MS		0.014	
BP-3		1 M acetate buffer (pH 5.0)	column Atlantis C18		0.197	
BP-8		incubation 37 °C, 8 h	(150 mm × 2.1 mm × 5 µm)		0.044	
4-OH-BP		LLE ethyl acetate			0.047	
BP-1	5 mL urine	Enzymatic hydrolysis			0.002	present study
BP-2		β-glucuronidase-*Helix pomatia*			0.002	
BP-3		1 M acetate buffer (pH 5.0)	UHPLC-ESI (+/−)-MS/MS		0.100	
BP-6		incubation 37 °C, overnight	column PFP Kinetex		0.002	
BP-7			(100 mm × 2.1 mm × 1.7 µm)		0.002	
BP-8		LLE ethyl acetate			0.005	
4-OH-BP		d-SPE Z-Sep			0.001	
4-MBC					0.050	
3-BC					0.050	

Note: italics—LOQ calculated from LOD × 3.

**Table 6:** Results of the analysis of 30 urine sample collected from Czech women (ng/mL in urine).

	BP-1	BP-2	BP-3	BP-6	BP-7	BP-8	4-OH-BP	4-MBC	3-BC
Positive samples (%)	100	20	90	7	33	20	100	0	0
Arithmetic mean *	19.5	<0.002	162	<0.001	<0.002	<0.005	0.711	-	-
Median *	2.24	<0.002	18.0	<0.001	<0.002	<0.005	0.351	-	-
5th percentile *	0.217	<0.002	0.661	<0.001	<0.002	<0.005	0.111	<0.100	<0.100
95th percentile *	112	<0.002	904	<0.001	<0.002	<0.005	2.21	<0.100	<0.100
Minimum value	0.083	0.019	1.58	0.031	0.015	0.010	0.060	<0.100	<0.100
Maximum value	150	1.13	1340	0.037	0.726	2.02	6.15	<0.100	<0.100

Note: * for results below LOQ and with more than 50% positive samples, value 1/2 of LOQ was used for calculation; values are rounded to three significant figures and maximum of three decimal places.

## Data Availability

The data presented in this study are available upon request from the corresponding author.

## Supplementary Materials

The following supporting information can be downloaded at: https://www.mdpi.com/article/10.3390/toxics12120837/s1, Table S1 Information about used certified standards; Table S2 Retention times and MS/MS parameters for determination of benzophenone and camphor UV filters; Table S3 Performance characteristics for OH-PAHs (except 6-OH-CHRY and 3-OH-BaP)-LLE with d-SPE; Table S4 Performance characteristics for 6-OH-CHRY and 3-OH-BaP-LLE with d-SPE.
