# Supplementary material for: Implementation of Sensitive Method for Determination of Benzophenone and Camphor UV Filters in Human Urine
Source: Toxics. 2024 Nov 21;12(12):837. doi: 10.3390/toxics12120837 (PMC11679341; doi:10.3390/toxics12120837)
Supplement: Supplementary file 1 [file toxics-12-00837-s001.zip › toxics-3284777-supplementary.pdf]

**Toxics**

**Electronic Supplementary Material**

# IMPLEMENTATION OF SENSITIVE METHOD FOR THE DETERMINATION OF BENZOPHENONE AND CAMPHOR UV FILTERS IN HUMAN URINE

Veronika Gomersall[1], Katerina Ciglova[1], Jana Pulkrabova[1*]

[1]University of Chemistry and Technology, Prague, Faculty of Food and Biochemical Technology, Department of Food Analysis and Nutrition, Technicka 3, 166 28 Prague 6, Czech Republic

*Corresponding author: Tel.: +420 220 443 272

E-mail: jana.pulkrabova@vscht.cz

| | | |
|---|---|---|
| ORCID ID: | Veronika Gomersall | 0000-0001-9954-9850 |
| | Katerina Ciglova | 0000-0001-5483-3209 |
| | Jana Pulkrabova | 0000-0003-1965-4672 |

**Table S1** Information about used certified standards

| Name | Abbreviation | CAS number | Purity (%) | Manufactures |
|---|---|---|---|---|
| benzophenone-1 | BP-1 | 131-56-6 | 99.7 | HPC Standards GmbH (Germany) |
| benzophenone-2 | BP-2 | 131-55-5 | 99.8 | |
| benzophenone-3 | BP-3 | 131-57-7 | 99.9 | |
| benzophenone-6 | BP-6 | 131-54-4 | 99.4 | |
| benzophenone-7 | BP-7 | 85-19-8 | 99.8 | |
| benzophenone-8 | BP-8 | 131-53-3 | 99.8 | |
| 4-hydroxy-benzophenone | 4-OH-BP | 1137-42-4 | 98.8 | |
| 3-benzylidene camphor | 3-BC | 15087-24-8 | ≥97.0 | |
| 3-(4-methylbenzylidene)-camphor | 4-MBC | 36861-47-9 | 99.8 | Sigma-Aldrich (USA) |
| benzophenone-3-$d_3$ | BP-3-$d_3$ | n.s. | 98.0 | Toronto Research Chemicals Inc. (Canada) |
| benzophenone-8-$d_3$ | BP-8-$d_3$ | n.s. | 98.0 | |
| 4-hydroxy-benzophenone-$d_4$ | 4-OH-BP-$d_4$ | 93496-64-1 | 98.0 | |
| 3-(4-methylbenzylidene)-camphor-$d_4$ | 4-MBC-$d_4$ | 1219806-41-3 | >99.5 | Chiron AS (Norway) |

*Note: n.s. …not specified*

**Table S2** Retention times and MS/MS parameters for determination of benzophenone and camphor UV filters

| Analyte | Retention time (min) | MRM transition | Ionisation mode | Declustering potential (V) | Entrance potential (V) | Collision energy (V) | Cell exit potential (V) |
|---|---|---|---|---|---|---|---|
| BP-1 | 5.83 | **213 > 135** | ESI- | -50 | -10 | -26 | -15 |
| | | 213 > 91 | ESI- | -20 | -10 | -34 | -11 |
| BP-2 | 4.80 | **245 > 135** | ESI- | -35 | -10 | -20 | -13 |
| | | 245 > 109 | ESI- | -35 | -10 | -26 | -13 |
| BP-3 | 7.15 | **227 > 211** | ESI- | -45 | -10 | -30 | -19 |
| | | 227 > 212 | ESI- | -45 | -10 | -22 | -19 |
| BP-6 | 7.21 | **273 > 123** | ESI- | -40 | -10 | -22 | -25 |
| | | 273 > 108 | ESI- | -40 | -10 | -46 | -13 |
| BP-7 | 6.91 | **231 > 121** | ESI- | -55 | -10 | -30 | -11 |
| | | 231 > 77 | ESI- | -50 | -10 | -36 | -55 |
| BP-8 | 6.01 | **243 > 123** | ESI- | -35 | -10 | -42 | -51 |
| | | 243 > 107 | ESI- | -35 | -10 | -42 | -51 |
| 4-OH-BP | 4.51 | **197 > 92** | ESI- | -65 | -10 | -40 | -11 |
| | | 197 > 120 | ESI- | -65 | -10 | -32 | -13 |
| 4-MBC | 7.99 | **255 > 165** | ESI+ | 116 | 10 | 59 | 16 |
| | | 255 > 105 | ESI+ | 116 | 10 | 37 | 12 |
| 3-BC | 7.35 | **241 > 91** | ESI+ | 51 | 10 | 41 | 8 |
| | | 241 > 165 | ESI+ | 51 | 10 | 61 | 16 |
| $(^2H)_4$-BP-2 | 4.78 | **249 > 137** | ESI- | -95 | -10 | -14 | -33 |
| | | 249 > 136 | ESI- | -95 | -10 | -14 | -17 |
| $(^2H)_3$-BP-3 | 7.13 | **230 > 212** | ESI- | -45 | -10 | -28 | -21 |
| | | 230 > 182 | ESI- | -45 | -10 | -70 | -21 |
| $(^2H)_3$-BP-8 | 6.01 | **246 > 126** | ESI- | -45 | -10 | -30 | -11 |
| | | 246 > 108 | ESI- | -45 | -10 | -30 | -45 |
| $(^2H)_4$-4-OH-BP | 4.49 | **201 > 124** | ESI- | -15 | -10 | -30 | -23 |
| | | 201 > 125 | ESI- | -15 | -10 | -30 | -17 |
| $(^2H)_4$-4-MBC | 7.96 | **259 > 216** | ESI+ | 101 | 10 | 27 | 22 |
| | | 259 > 108 | ESI+ | 101 | 10 | 37 | 10 |

*Note: **bold**… transitions for quantification*

**Table S3** Performance characteristics for OH-PAHs (except 6-OH-CHRY and 3-OH-BaP) – LLE with d-SPE

| | 2-OH-NAP | 1-OH-NAP | 2-OH-FLUO | 2-OH-PHEN | 3-OH-PHEN | 1-OH-PHEN | 9-OH-PHEN | 4-OH-PHEN | 1-OH-PYR |
|---|---|---|---|---|---|---|---|---|---|
| **ISTD** | **2-OH-NAP-d7** | **1-OH-NAP-d7** | **2-OH-FLUO-d9** | **2-OH-PHEN-d9** | **3-OH-PHEN-d9** | **1-OH-PHEN-d9** | **9-OH-PHEN-d8** | **9-OH-PHEN-d8** | **1-OH-PYR-d9** |
| **LOQ** ng/mL urine | **0.001** | **0.002** | **0.001** | **0.001** | **0.001** | **0.002** | **0.005** | **0.001** | **0.002** |
| **SRM 3673** | | | | | | | | | |
| Certified value (ng/mL urine) | 1.32 ± 0.03 | 207 ± 33 | 0.105 ± 0.007 | 0.0242 ± 0.0042 | 0.0271 ± 0.0014 | 0.0479 ± 0.0074 | 0.0114 ± 0.0009 | 0.0102 ± 0.0010 | 0.0299 ± 0.0018 |
| Measured value (ng/mL urine) | 1.43 ± 0.09 | 146 ± 12 | 0.106 ± 0.0160 | 0.0267 ± 0.0041 | 0.0278 ±0.003 | 0.0514 ± 0.0063 | 0.0134 ± 0.0015 | 0.0088 ± 0.0005 | 0.0307 ± 0.0023 |
| Recovery (%) | 108 | 71 | 101 | 110 | 103 | 107 | 118 | 86 | 103 |
| RSD (%) | 6 | 8 | 15 | 15 | 11 | 12 | 11 | 5 | 8 |

*Note: [a]…uncertainty was calculated as a standard deviation; ISTD… isotopically labelled standards used as surrogates; LOQ…method limit of quantification; RSD…repeatability expressed as relative standard deviation.*

**Table S4** Performance characteristics for 6-OH-CHRY and 3-OH-BaP – LLE with d-SPE

| | 6-OH-CHRY | 3-OH-BaP |
|---|---|---|
| **ISTD** | **1-OH-PYR-d9** | **3-OH-BaP-d11** |
| **LOQ** ng/mL urine | **0.005** | **0.050** |
| **Level 0.05 ng/mL** | | |
| Recovery (%) | 95 | - |
| RSD (%) | 9 | - |
| **Level 0.5 ng/mL** | | |
| Recovery (%) | 97 | 99 |
| RSD (%) | 9 | 5 |

*Note: ISTD… isotopically labelled standards used as surrogates; LOQ…method limit of quantification; RSD…repeatability expressed as relative standard deviatio*
